# Characterization of semi-arid Chadian sweet sorghum accessions as potential sources for sugar and ethanol production

**DOI:** 10.1038/s41598-020-71506-9

**Published:** 2020-09-11

**Authors:** Gapili Naoura, Yves Emendack, Nébié Baloua, Kirsten vom Brocke, Mahamat Alhabib Hassan, Nerbewende Sawadogo, Amos Doyam Nodjasse, Reoungal Djinodji, Gilles Trouche, Haydee Echevarria Laza

**Affiliations:** 1 Institut Tchadien de Recherche Agronomique Pour le Développement (ITRAD), B.P. 5400, N’Djaména, Chad; 2 Cropping Systems Research Laboratory, USDA-ARS, Lubbock, TX 79415 USA; 3 International Crops Research Institute for the Semi-Arid Tropics (ICRISAT), Bamako, Mali; 4 AGAP, Univ Montpellier, CIRAD, INRA, Montpellier SupAgro, University of Montpellier, 34090 Montpellier, France; 5 Laboratoire Biosciences, Équipe Génétique et Amélioration des Plantes, Université Joseph KI-ZERBO, 03 BP 7021, Ouagadougou 03, Burkina Faso; 6 Department of Plant and Soil Sciences, Texas Tech University, Lubbock, TX 79409 USA; 7 CIRAD, UMR AGAP, 34398 Montpellier, France

**Keywords:** Plant sciences, Plant breeding

## Abstract

Sweet sorghum (*Sorghum bicolor* (L.) Moench) is an important crop in Chad that plays an economic role in the countryside were stalks are produced mainly for human consumption without any processing. Unfortunately, very little information exists on its genetic diversity and brix content. Studies performed in 2014 and 2015 showed that there were significant variations (*p* < 0.001) for all assessed quantitative traits. Potential grain yield (0.12–1.67 t ha^−1^), days to 50% flowering (68.3–126.3 days), and plant height (128.9–298.3 cm) were among traits that exhibited broader variability. Brix content range from 5.5 to 16.7% across accessions, was positively correlated to stalk diameter and plant height, but negatively correlated to moisture content in fresh stalk and potential grain yield. Fresh stalk yield range from 16.8 to 115.7 Mg ha^−1^, with a mean value of 58.3 Mg ha^−1^ across accession. Moisture content in fresh stalk range from 33.7 to 74.4% but was negatively correlated to fresh stalk yield. Potential sugar yield range from 0.5 to 5.3 Mg ha^−1^ across accession with an average of 2.2 Mg ha^−1^. Theoretical ethanol yield range from 279.5 to 3,101.2 L ha^−1^ across accession with an average of 1,266.3 L ha^−1^ which is significantly higher than values reported under similar semiarid conditions. Overall, grain yields were comparatively low. However, two accessions had grain yield of more than 1.5 t ha^−1^; which is greater than the average 1.0 t ha^−1^ for local grain sorghum varieties in Chad. These could have multi-purpose uses; grains, sugar and bioenergy production.

## Introduction

Sweet sorghum [*Sorghum bicolor* (L.) Moench] is an annual, seed-propagated C4 grass that derived its name from the high concentration of soluble sugars (a mixture of sucrose, glucose, and fructose) contained in its tall, juicy stalks^22,26,38^. In Chad, sweet sorghum also commonly known as sugar sorghum, is cultivated by several farmers on small areas or sprinkled in the grain sorghum fields. It is also cultivated around village huts for consumption by children while their parents are away to their farms out of the villages^13^. The national agricultural statistics of the ministry in charge of agricultural production in Chad does not take sweet sorghum into account, thus information on the national production, the extent of its cultivation and genetic diversity are not available. The cultivation of sweet sorghum had been abandoned by farmers, threatening its genetic resources. Recent regained interest in the sale and consumption of sweet sorghum stalk in local markets has led to a boost in cultivation by small scale farmers^13^.

Most of the world’s ethanol production is obtained from two major crops: corn and sugarcane^6^. Sweet sorghum offers one of the best plant-based bioethanol productions from its sugary stalk^7^ and is considered a potential bioenergy crop throughout most of the tropical and temperate zones of the world, and it is also a leading contender for biofuel production in the southern United States^46^. Numerous studies have been performed to assess the agronomic performance and yields of juice, sugar and ethanol of sweet sorghum^2,3,6,10,11,12,18,20,32,44^. Compared to other bioenergy crops such as corn, wheat, sugarcane, sugar beet, cassava, sweet potato; sweet sorghum is drought tolerant, requires lower quantity of water (e.g. 1/3 of sugarcane, 1/2 of corn) and fertilization inputs, has tolerance to salinity, i.e. can be grown in marginal regions that are not commonly used for crop production and it also has lower greenhouse gas emissions on a life-cycle basis^1,2,9^.

Sweet sorghum stalk is used for the production of food grade syrup, alcohol, and even chewed fresh in Brazil and India^17^. With its high sucrose content, the stalk is fermented for the production of bioethanol^7,29^ and can yield up to 8,000 L ha^−1^ of ethanol which is approximately twice the ethanol yield of corn and 30% greater than the average produced from sugarcane^24^. In 2019, the global fuel ethanol production was 110.1 billion liters, the two largest producers being the U.S. and Brazil with 59.8 and 32.6 billion litters respectively^45^.

There are about 4,000 sweet sorghum cultivars distributed through the world^35^. In Chad, sugar sorghum is grown for human consumption without any prior processing. Stalks are sold on the roadsides in villages or transported by trucks to large urban centers. In some rural areas in southern Chad, sugar sorghum is the main source of revenue and economic sustainability^13^. Despite the importance of sweet sorghum in Chad’s human nutrition, few researches have evaluated the genetic diversity of the Chadian accessions. A study conducted as part of the sweet sorghum collection survey revealed a large diversity in the cultivars grown by farmers^13^.

The main purposes of this study were to: (1) determine the agro-morphological and phenological diversity in traits associated with yields of juice and ethanol of sweet sorghum accessions grown in the Sudanese zone of Chad, (2) identify high-performing cultivars which could be used for genetic improvement of grain sorghum accessions grown and sold in Chad and (3) identify high performing accessions with respect to ethanol production which could be beneficial to the global bioenergy production research especially in similar ecological regions.

## Materials and methods

### Plant materials

The plant material consisted of 105 landraces from the Bébédjia research station. These were local varieties from a prospecting collection carried out in the Sudanese zone (comprising Logone Occidental, Logone Oriental, Mandoul, Mayo Kebbi West, Mayo Kebbi East, Moyen Chari, and Tandjilé) of Chad in 2012^13^ (Table 1). Five improved sweet sorghum varieties from ICRISAT Mali, were used as checks (see Supplementary Table 1 for accessions numbers and names of varieties).

**Table 1:** Rainfall, regional distribution, and origins of sweet sorghum accessions used in study.

Regions	Departments	Number of villages	Number of accessions	Annual rainfall (mm)	
				2015	2014
Logone Occ	Lac Houé	1	1	951	1,100
	Dodjé	2	6	1,278	1,077
	Ngourkosso	2	2	905	934
	*Total*	*5*	*9*		
Logone Ori	Mont de Lam	3	11	1639	1,281
	Kou Est	1	4	1,432	1,298
	Kou Ouest	2	4	1,229	920
	Nya-Pendé	3	10	1,428	1,159
	Pendé	2	10	1,240	951
	*Total*	*11*	*39*		
Mandoul	Mandoul Occ	2	4	914	881
	Mandoul Ori	4	8	1,014	851
	*Total*	*6*	*12*		
Mayo-Kebbi E	Mayo Boneye	*2*	*7*	*874*	*712*
Mayo-Kebbi W	Mayo Dallah	5	11	1,006	860
	Lac Léré	3	3	778	896
	*Total*	*8*	*14*		
Moyen Chari	Barh Kôh	*1*	*5*	1,157	1,062
Tandjilé	Tandjilé W	1	3	1,017	896
	Tandjilé E	8	16	1,069	925
	*Total*	*9*	*19*		
	CHAD	41	105		
	ICRISAT		5		
	TOTAL		110		

### Experimental design

Field experiments were conducted at the ITRAD Research Centre in Bébédjia (9° 55′ N Latitude North and 15° 8′ Longitude East), during the growing season from April to October for two consecutive years; 2014 and 2015, on a poorly desaturated sandy clay soil type. The experimental design was an α-lattice with three replications, each of which was subdivided into twelve plots of ten lines. Seeds from each accession were sown in holes on 10 m long lines, with 0.7 m spacing between lines and 0.3 m between seed holes. 100 kg ha^−1^ of NPK fertilizer was applied 15 days after planting.

### Data collection

Seasonal rainfall distribution was measured every 10 days through the growing season in both years. Plant data were collected over the entire period of plant development, from emergence to maturity and after harvesting. As qualitative characters, the color and vigor of seedling were noted at emergence; seedling vigor rated from 1 to 9 based on stem size, leaf thickness and length of seedlings. 1 indicated poor vigor, 3 weak vigor, 5 fair vigor, 7 good vigor, and 9 excellent vigor. Panicle compactness, color of leaf midrib, grain color, presence of dimple, glume color, and glume hairiness were determined at maturity. The botanical race was determined in the field according to^15^ and confirmed in the laboratory. Virtuosity was estimated according to the BONO scale (0–4): 0 for totally floury grains, 1 for rather floury grains, 2 for vitreous grains (at 50% vitreous), 3 for rather vitreous grains (more than 50% vitreous), and 4 for entirely vitreous grains.

Quantitative characteristics such as number of days to heading (NDH) and number of days to flowering (NDF; 50% flowering in accession) were determined from planting. At the hard dough stage, the perultimate leaf length (PLL; base of leaf to leaf tip), perultimate leaf width (PLW; at widest part of leaf), plant height (PHT, base of plant to tip of panicle), number of internodes (NIN), stem diameter (SDI; at fourth internode under panicle), panicle length (PAL; base to tip of panicle), panicle width (PAW; at broadest part of panicle), and internode length (INL; average of third and fifth internode under panicle) were determined from 10 randomly selected plants per accession. At maturity, main panicles of the 10 plants were harvested, air dried to constant weight for at least 10 days, and weighed to determine the main panicle (PWT). Panicles were thrashed and grains weighed to determine the weight of grains of the main panicle (PGW). The potential yield (PYI) was obtained by multiplying the average PGW from the 10 main panicles by the number of seedlings per hectare for each accession. Thousand grains weight (TGW) was also determined.

Brix content was measured from internodes using a hand-held refractometer (Master, Atago, Japan). Brix content is influenced significantly by the positions of the internodes^39^. For this study the brix content was measured from the fourth and sixth internodes below the panicle. To determine the fresh and dry weight of stalk, the 10 randomly selected plants were (harvested at 5 cm above the soil surface) were stripped of leaves and panicles, and the stalks were weighed for fresh stalk weight (FSW). The stalks were air dried in the shade for at least 10 days until they reached constant weight for three consecutive days, at which the field dried stalk weight (DSW) was measured. Moisture content was determined as the percent difference between FSW and DSW. The fresh stalk yield (FSY) and dry stalk yield (DSY) were calculated as average of FSW and DSW respectively multiplied by number of plants per hectare^10,39^.

Juice and sugar yields were calculated according to^43^:

$$\begin{aligned} & {\text{CSY}} = \left( {{\text{FSY}} - {\text{DSY}}} \right) \times {\text{Brix }} \times 0.75 \\ & {\text{JCY}}\,\left( {80\% \,{\text{extracted}}} \right) = \left[ {{\text{FSY}} - \left( {{\text{DSY}} - {\text{CSY}}} \right)} \right] \times 0.8 \\ & {\text{SGY}} = {\text{JCY}} \times {\text{Brix}} \times 0.75 \\ \end{aligned}$$

where CSY is conservative sugar yield (Mg ha^−1^), FSY is fresh stalk yield (Mg ha^−1^), DSY is dry stalk yield (Mg ha^−1^), JCY is juice yield (Mg ha^−1^), and SGY is sugar yield (Mg ha^−1^).

Sugar concentration of juice (SCJ) was determined as 75% of brix expressed in g kg^−1^ sugar juice^35^:

$${\text{SCJ}}\,\left( {{\text{g}}\,{\text{Kg}}^{ - 1} } \right) = 0.75 \times {\text{Brix}}$$

Theoretical ethanol yield (TEY, L ha^−1^) from extracted juice was calculated as sugar yield (kg ha^−1^) multiplied by a conversion factor of 0.581 L kg^−1^ sugar^41^:

$${\text{TEY}}\,\left( {{\text{L}}\,{\text{ha}}^{ - 1} } \right) = {\text{CSY }} \times {0}{\text{.581}}$$

Total soluble sugar (y, %) was estimated using equation by^23^:

$${\text{y}}\,\left( \% \right) = 0.8111{\text{x}}{-}0.37285$$

where x is the Brix of stalk juice.

### Data analysis

Analysis of variance (ANOVA) and Newman-Keuls test were performed to determine if the average of the quantitative characteristics varied significantly (at 5.0, 1.0, and 0.1% probability thresholds) between accessions. Some parametric distributions were calculated to see the dispersion of the values of the characteristics based on the average, the minimum, the maximum and the coefficient of variation. For all characteristics, genetic variabilities were estimated from the components of the analysis of variance, and broad heritability (H^2^) was calculated using the formulas by^16^. Bivariate analysis was carried out, using the Pearson correlation coefficient to see the link between two characteristics. Multivariate analyses were performed through Principal Component Analysis (PCA) to highlight uncorrelated characters, which were used to build the dendrogram from the Hierarchical Ascending Classification (HAC). Then the Discriminant Factor Analysis (DFA) was carried out to characterize the group from the HAC.

## Results

### Seasonal rainfall distribution

**Figure 1:**
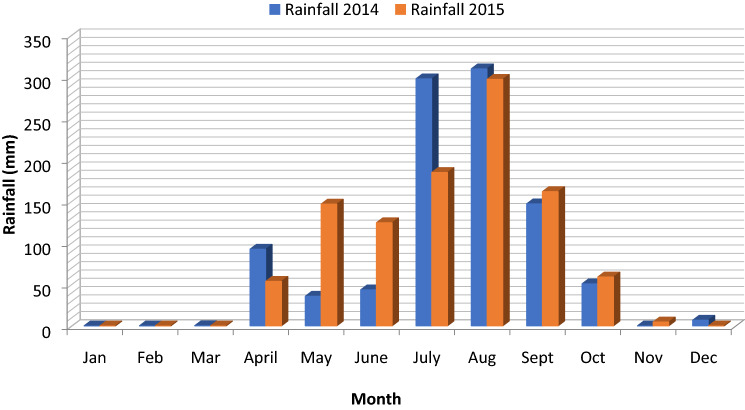
Season rainfall distribution measured during the 2014 and 2015 sweet sorghum growing season at the ITRAD Research Centre in Bébédjia, Chad.

Total season rainfall (Fig. 1) from planting to harvest was lower in 2014 (908.2 mm) compared to 2015 (1,098.5 mm). However, seasonal rainfall distribution showed crops receiving similar amount of rainfall (609.6 vs. 605.7) during the peak of crop growth and development in 2014 and 2015 respectively.

### Variability in agro-morphological traits

Analysis of qualitative traits (Table 2) showed a great diversity among sweet sorghum accessions from Chad. Generally, 64% of seedlings were green and 36% violet, and these were accession specific. Seedling vigor ratings showed frequencies of 31.4, 32.9, and 21.2% for low, fair, and good vigor respectively. The color of the leaf midrib (a phenotypic characteristic of sweet sorghum) was green for 69.5% and white for 29.5% of the accessions. Most accessions had loose panicles (51.9%) with straw colored glumes (80.9%), red seed (84.5%), often very floury in vitreousity (69.7%) and mainly from the caudatum (64.55%) or bicolor (35.45%) race.

**Table 2:** Modeling for the qualitative characteristics of sweet sorghum in semi-arid Chad.

Characteristics	Modality	Frequency (%)
Color of seedling	Green	64.2
	Violet	35.7
Vigor of seedling	Excellent	7.3
	Good	21.2
	Fair	32.9
	Weak	31.4
	Poor	7.3
Color of leaf midrib	Green	69.7
	White	29.5
	Black	11.8
	Yellow	0.2
Glume color	Straw	80.9
	Red	7.3
Seed color	Red	84.5
	White	15.5
Vitreousity	Floury	69.7
	Vitreous	30.3
Panicle	Loose	51.9
	Semi-compact	32.6
	Compact	15.5
Botanical race	*Caudatum*	64.6
	*Bicolor*	35.4
Glume hairiness	Minimal	55.8
	Partial	36.4
	Hairy	7.8
Grain dimple	Absent	52.7
	Present	47.3

The analysis of variance (Table 3) showed highly significant differences (*p* < *0.001*) between accessions in all assessed quantitative agronomic characteristics. The coefficients of variation (CV) for most characters was above 30%, reflecting variability amongst accessions. Heritability (H^2^) range from 58.6 to 98.8%. The strongest agronomic heritability (H^2^ > 80%) were recorded for number of days to flowering (95.6), number of days to heading (95.5), plant height (94.9), number of internodes (94.5), internode length (90.2), panicle length (90.2), stalk diameter (83.9), dry stalk weight (82.1), and perultimate leaf width (81.0). The lowest heritability was scored for potential yield (64.8), panicle grain weight (64.8), panicle weight (61.5) and panicle width (58.6).

**Table 3:** Variance of agronomic characteristics of Chadian sweet sorghum accessions.

Characteristics	Minima	Maxima	Average	CV (%)	F-value	H^2^ (%)
PHT (cm)	128.9	298.3	232.2 ± 47.9	20.6	17.1***	94.9
SDI (cm)	1.0	2.2	1.73 ± 0.3	17.9	5.7***	83.9
PLW (cm)	5.4	11.4	8.2 ± 1.1	13.3	4.4***	81.0
PAW (cm)	4.7	13.0	7.4 ± 1.4	31.7	2.3***	58.6
INL (cm)	14.4	34.9	21.6 ± 3.8	36.2	9.2***	90.2
PLL (cm)	46.0	83.3	68.3 ± 7.1	55.9	3.3***	74.4
PAL (cm)	10.9	35.9	24.8 ± 4.6	45.4	8.9***	90.2
NIN	4.9	16.2	10.3 ± 3.2	43.5	15.1***	94.5
NHD (days)	68.3	126.3	94.2 ± 15.2	16.0	19.8***	95.5
NFW (days)	68.3	126.3	95.1 ± 15.1	16.0	20.3***	95.6
PWT (g)	160.0	1,266.7	502.7 ± 215.8	42.9	2.4***	61.5
PGW (g)	60.0	833.3	272.4 ± 136.7	50.2	2.7***	64.8
PYI (t ha^−1^)	0.1	1.67	0.54 ± 0.27	50.2	2.7***	64.8
TGW (g)	12.7	33.3	21.39 ± 4.31	20.2	–	–
FSW (g)	116.7	783.3	403.8 ± 150	37.1	4.6***	79.8
DSW (g)	44.3	511.1	217.3 ± 101.9	46.9	5.0***	82.1

***Indicates significance at *p* < *0.001*, CV; coefficient of variation, H^2^; heritability, PHT; plant height, SDI; stalk diameter, PLW; perultimate leaf width, PAW; panicle width, INL; internode length, PLL; perultimate leaf length, PAL; panicle length, NIN; number of internodes, NHD; number of days to heading, NFW; number of days to flowering, PWT; panicle weight, PGW; panicle grain weight, PYI; potential yield, TGW; 1,000-grain weight, FSW; fresh stalk weight, DSW; field dried stalk weight.

Other traits (see Supplementary Table 1) showed significant variability with some Chadian accessions performing better than the ICRISAT checks. Compared to the average of ICRISAT checks, 22 Chadian accessions were taller, 63 had shorter days to heading, 54 had shorter days to flowering, 46 had higher number of internodes, 20 had longer internode lengths, 16 had longer perultimate leaf, 7 had wider perultimate leaf, 19 had longer panicle, 48 with wider panicle, 41 with higher fresh stalk biomass, 74 with higher dry stalk biomass, and 33 with broader stem diameter. Five of the 105 accessions screened had potential grain yields equal to or higher than the average 1.0 t ha^−1^ of grain sorghum production in semi-arid Chad.

All assessed quantitative characteristics linked to sugar production showed significant variability (*p* < 0.001) within and when compared to ICRISAT checks (Table 4). The coefficient of variation was high (CV > 30%) for most characteristics except for Brix (19.8%), total soluble sugar (20.6%) and the sugar concentration on juice (20.1%). The heritability was very high for all assessed characteristics except for the Juice yield (59.6%) and moisture content of fresh stalk weight (57.6%). Heritability was strongest for brix (98.8%), sugar concentration of juice (98.8%) and total soluble sugar (98.8%), followed by dry stalk yield (82.1%) and then fresh stalk yield (79.8%).

**Table 4:** Analysis of variance of twelve characteristics determining sugar and ethanol production in 105 accessions of semi-arid Chadian sweet sorghum.

Characteristics	Minima	Maxima	Average	CV (%)	F-value	H^2^ (%)
MFS (%)	33.7	74.4	48.7 ± 8.7	30.3	2.3***	57.6
FSY (Mg ha^−1^)	16.7	115.7	58.3 ± 22.1	38.0	4.6***	79.8
DSY (Mg ha^−1^)	6.5	75.5	31.3 ± 14.7	47.1	5.0***	82.1
JCY (Mg ha^−1^)	7.3	43.0	23.6 ± 7.7	32.7	2.4***	59.6
CSY (Mg ha^−1^)	0.6	5.9	2.5 ± 1.1	43.4	4.0***	75.7
SGY (Mg ha^−1^)	0.5	5.3	2.2 ± 1.0	44.5	4.2***	76.7
SCJ (g kg^−1^)	41.3	125.0	89.7 ± 18	20.1	86.8***	98.8
y (%)	4.1	13.1	9.3 ± 1.9	20.6	86.8***	98.8
TEY (L ha^−1^)	279.5	3,101.2	1,266.3 ± 563	44.5	4.2***	76.7
Brix (%)	5.5	16.7	11.9 ± 2.4	19.8	86.8***	98.8

***Indicates significance at *p* < *0.001*, CV; Coefficient of variation, MFS; moisture content of fresh stalk, FSY; fresh stalk yield, DSY; field dried stalk yield, CSY; conservative sugar yield, JCY; juice yield, SGY; sugar yield, SCJ; sugar concentration of juice, TEY; Theoretical ethanol yield, y; total soluble sugar. ± standard error.

The fresh stalk yield range from 16.75 to 115.7 Mg ha^−1^ and the dry stalk yield from 6.54 to 75.5 Mg ha^−1^, giving a moisture content range of 33.7 to 74.4%, with an average of 48.7%. The juice yield for 80% of the sugar extracted (represented the sugar for the stalk) range from 7.34 to 43 Mg ha^−1^ with an average of 23.6 Mg ha^−1^. The brix content range from 5.5 to 16.7% and the total soluble sugar range from 4.1 to 13.1 Mg ha^−1^ with an average of 9.3 Mg ha^−1^. The potential sugar yield range from 0.5 to 5.3 Mg ha^−1^ with an average of 2.2 Mg ha^−1^ and the theoretical ethanol yield range from 279.5 to 3,101.2 L ha^−1^ with average of 1,266 L ha^−1^.

Compared (see Supplementary Table 1) to the ICRISAT checks (F60, IS23525, IS23536, IS23541, and IS23574), 37 Chadian accessions had more brix, 39 more fresh stalk yield, 31 more dry stalk yield, 35 more juice yield, 32 more conservative sugar yield, 19 more juice yield, 12 more soluble sugars, and 22 more theoretical ethanol yield; with 10 accessions (ecotypes numbers 21, 46, 64, 66, 72, 80, 81, 82, 130, and 137) having significantly higher theoretical ethanol yield than the best ICRISAT variety (IS23541; TEY of 1695 L ha^−1^).

### Correlation analysis amongst characteristics

The Pearson correlation matrix (Table 5) shows the strength of the relationship between quantitative traits based on 5% (*p* < *0.05*), 1% (*p* < *0.01*) and 0.1% (*p* < *0.001*) probability levels. Brix content was significantly and positively correlated with theoretical ethanol yield (r = 0.75, *p* < *0.001*), potential sugar yield (r = 0.75, *p* < *0.001*), fresh stalk yield (r = 0.52, *p* < *0.001*), and dry stalk yield (r = 0.54, *p* < *0.001*). Moisture content in fresh stalk was negatively correlated to brix content (r =  − 0.47, *p* < *0.001*). Brix content was negatively correlated to thousand grain weight (r =  − 0.20, *p* < *0.05*) and potential grain yield (r =  − 0.26, *p* < *0.05*).

**Table 5:** Correlation coefficient for agro-morphological and phenotypic characteristics of 105 semi-arid Chadian sweet sorghum accessions.

Traits	PHT	PLL	PAL	NFW	SDI	NIN	Brix	FSY	DSY	JCY	SGY	TEY	MFS	TGW
PLL	0.48***													
PAL	0.72***	0.44***												
NFW	0.70***	0.50***	0.32											
SDI	0.65***	0.59***	0.41***	0.81***										
NIN	0.82***	0.49***	0.45***	0.92***	0.86***									
Brix	0.50***	0.14	0.32***	0.37***	0.37***	0.43***								
FSY	0.76***	0.50***	0.45***	0.79***	0.81***	0.81***	0.52***							
DSY	0.78***	0.52***	0.42***	0.82***	0.82***	0.85***	0.54***	0.97***						
JCY	0.64***	0.40***	0.44***	0.63***	0.69***	0.65***	0.46***	0.92***	0.80***					
SGY	0.65***	0.31	0.43***	0.60***	0.66***	0.64***	0.75***	0.88***	0.80***	0.92***				
TEY	0.65***	0.31	0.43***	0.60***	0.66***	0.64***	0.75***	0.88***	0.80***	0.92***	1.00***			
MFS	− 0.73***	− 0.54***	− 0.37***	− 0.73***	− 0.64***	− 0.77***	− 0.47***	− 0.68***	− 0.80***	− 0.39***	− 0.45***	− 0.45***		
TGW	− 0.34***	− 0.14	− 0.12	− 0.31**	− 0.21*	− 0.31**	− 0.20*	− 0.34***	− 0.33***	− 0.29**	− 0.27**	− 0.27**	0.32**	
PYI	− 0.24*	0.09	− 0.23*	− 0.03	0.06	− 0.06	− 0.26*	− 0.20*	− 0.17	− 0.23*	− 0.24*	− 0.24*	0.19	0.30**

*,**,*** Significance at *p* < *0.05*, *p* < *0.01*, *p* < *0.001*; PHT: plant height; PLL: perultimate leaf length; PAL: panicle length; NFW: number of days to flowering; SDI: stem diameter; NIN: number of internodes; FSY: fresh stalk yield; DSY: field dried stalk yield; JY: juice yield; SY: sugar yield; TEY: theoretical ethanol yield, MFS: moisture content in fresh stalk; TGW: 1,000-grain weight; PYI: potential yield.

Greater theoretical ethanol yields were correlated to taller plants (r = 0.65, *p* < *0.001*), thicker stems (r = 0.66, *p* < *0.001*), number of internodes internodes (r = 0.64, *p* < *0.001*), longer panicles, fresh stalk (r = 0.88, *p* < *0.001*) and dry stalk (r = 0.80, *p* < *0.001*) yields, number of days to flowering (r = 0.60, *p* < *0.001*). Moisture content in fresh stalk had a negative correlation to all assessed characteristics except thousand grain weight (r = 0.32, *p* < *0.01*).

Late maturity correlated positively to larger stem diameter, number of internodes, fresh and dry stalk yields, juice and sugar yields but negatively to thousand grain weight, with no significant correlation to potential grain yield. Taller plants with longer panicles, high brix content, high fresh and field dried stalk yields, and high juice and sugar yields were negatively related to potential grain yield.

### Cluster analysis and diversity organization

**Figure 2:**
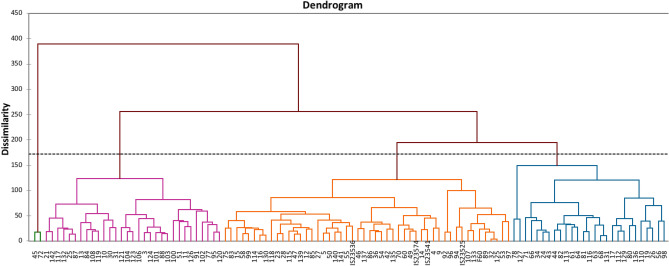
Clustering of 110 sweet sorghum accessions into 4 groups using the standardized squared Euclidean distance of Ward’s hierarchical clustering method.

Hierarchical clustering, using Ward’s method and truncation on level 175, grouped the 110 sweet sorghum accessions into four major groups (Fig. 2). Group 1 was composed of 31 accessions and was further subdivided into 2 sub-groups; 1a (13 accessions) and 1b (18 accessions). Group 2 had the largest number of accessions (50) and was subdivided into 2 sub-groups; 2c (38 accessions) and 2d (12 accessions). Group 3 consisted of 27 accessions divided into 2 sub-groups; 3e (2 accessions) and 3f. (25 accessions). Group 4 was composed of 2 accessions. The Mahalanobis distance obtained through the Discriminant Factor Analysis (DFA) showed long distance between all the groups (*p* < *0.001* and *p* < *0.01*), which signified that the four groups were quite distinct (Table 6).

**Table 6:** Distances of Mahalanobis and statistical significant of Fisher.

	Group 1	Group 2	Group 3
Group 2	14.0***		
Group 3	49.7***	12.2***	
Group 4	117.5***	52.5***	15.7**

**,***indicates significance at *p* < *0.01* and *p* < *0.*001 respectively.

The mean values of the accessions in each cluster are given in Table 7, obtained through the Discriminant Factor Analysis (DFA). Group 1 accessions were characterized as short, early flowering, with lowest fresh stalk weight and the lowest average brix content (10.7%). Groups 2 and 3 showed mean values of all characteristics studied, with an average brix value of 11.6% and 13.7% respectively. The two accessions in group 4 were characterized as the tallest plants, late flowering, with the heaviest fresh stalk weight and the highest average brix value (15.2%).

**Table 7:** Comparison of agro-morphological and phenotypic traits of four groups of Chadian sweet sorghum derived from Ward’s hierarchical clustering.

Characteristics	Group 1	Group 2	Group 3	Group 4
PHT (cm)	213.9 ± 46	230.7 ± 47.7	251.5 ± 42.0	295.3 ± 1.6
PLL (cm)	68 ± 6.9	66.9 ± 7.4	70.7 ± 6.1	75.3 ± 1.8
PLW (cm)	7.8 ± 1.0	8.1 ± 1.2	8.7 ± 0.9	8.9 ± 0.7
PAL (cm)	24.5 ± 3.9	23.9 ± 3.9	26.4 ± 5.9	30.5 ± 4
PAW (cm)	6.9 ± 1.5	7.4 ± 1.4	7.9 ± 1.4	7.9 ± 0.5
INL (cm)	22.39 ± 4.1	21.2 ± 3.5	21.4 ± 4.2	21.9 ± 3.2
NFW (days)	88.2 ± 11.2	94.2 ± 15.8	103.4 ± 13.7	109.3 ± 8
FSW (g)	223.6 ± 53.9	403.7 ± 46.0	583.3 ± 63.5	777.8 ± 7.9
Brix (%)	10.7 ± 2.7	11.64 ± 1.8	13.7 ± 1.7	15.2 ± 2.1
Number of accessions	31	50	27	2

PHT: plant height; PLL: perultimate leaf length; PLW: perultimate leaf width; PAL: panicle length; PAW: panicle width; INL: internode length; NFW: number of days to flowering; FSW: fresh stalk weight. ± standard error.

The structuring of the individuals in each group (Table 8) shows a distribution of accessions based on their regions of origin. Group 2 represented all the regions of origin of the accessions. On the other hand, in group 1, the accessions of the Moyen Chari region were not represented. In group 3 accessions of Mayo Kebbi Ouest were not represented. Finally, in group 4, only the accessions of the Oriental Logone were found. The accessions from ICRISAT (see Supplementary Table 1: F60, IS23525, IS23536, IS23541, and IS23574) were found only in group 2.

**Table 8:** Regional distribution of the accessions of Chadian sweet sorghum following Ward’s hierarchical clustering into four major groups.

Regions	Group 1	Group 2	Group 3	Group 4	Total
Tandjilé	9	8	2	0	19
Mayo Kebbi Est	3	4	0	0	7
Mayo Kebbi Ouest	4	5	5	0	14
Logone Oriental	10	13	14	2	39
Logone Occidental	3	6	1	0	9
Mandoul	2	6	4	0	12
Moyen Chari	0	4	1	0	5
Accession from ICRISAT	0	5	0	0	5
Total	31	50	27	2	110

### Promising genotypes for sweet sorghum improvement program in Chad

This multi-year evaluation of the 105 Chadian sweet sorghum accessions showed higher performance in juice and sugar yields of 10 accession compared to the 5 improved sweet sorghum from ICRISAT considered as high performers (Table 9). The "*Balnda*" accession had 16.7% brix, 42.6 Mg ha^−1^ juice yield, 5.3 Mg ha^−1^ potential sugar yield, and a theoretical ethanol yield of 3,101.2 L ha^−1^. It was followed by the accession "Sian *Guebeuh*" with 2,712.2 L ha^−1^ of theoretical ethanol yield, and "Var137" with 2,669.5 L ha^−1^ of theoretical ethanol yield. The "Zimikaye Combole" accession, despite its 13.7% brix, achieved a high sugar yield (4.4 Mg ha^−1^) and ethanol yield (2,570 L ha^−1^). The best ICRISAT varieties was IS23541 with 14.1% brix, 27.4 Mg ha^−1^ juice yield, 2.9 Mg ha^−1^ potential sugar yield, and 1695.0 L ha^−1^ theoretical ethanol yield.

**Table 9:** Compositional characteristics of 10 promising genotypes of Chadian sweet sorghum compared to ICRISAT checks (IS: 23,541, 23,574, 23,525, 23,536, and F60).

Accessions Name	Brix	FSY	DSY	JCY	CSY	SGY	SCJ	y	TEY
(Ecotype #)	(%)	(Mg ha^−1^)					(g kg^−1^)	(%)	(L ha^−1^)
"Balnda" (66)	16.7	80.0	32.8	42.6	5.9	5.3	166.7	13.1	3,101.2
"Sian Guebeuh" (21)	15.0	104.3	57.8	41.4	5.2	4.7	150.0	11.8	2,712.2
"Var137" (137)	15.7	97.8	54.2	39.0	5.1	4.6	156.7	12.3	2,669.5
"Zimikay" (72)	13.7	115.7	67.0	43.0	5.0	4.4	136.7	10.7	2,570.0
"Chian Woua" (46)	16.7	114.1	75.5	34.7	4.8	4.3	166.7	13.1	2,530.5
"Kadbal" (80)	14.3	96.0	52.9	38.2	4.6	4.1	143.3	11.3	2,393.1
"Kad bel hym" (81)	15.3	82.5	47.2	31.5	4.1	3.6	153.3	12.1	2,111.0
"Kad Nda" (82)	15.3	80.5	46.3	30.5	3.9	3.5	153.3	12.1	2047.2
"Bagnadé" (64)	15.7	77.6	44.3	29.7	3.9	3.5	156.7	12.3	2036.1
"Syan Teigne" (130)	16.0	91.1	58.7	29.1	3.9	3.5	160.0	12.6	2032.6
IS23541	14.1	71.4	40.4	27.4	3.3	2.9	141.1	11.1	1695.0
IS23574	9.7	66.5	38.8	23.8	2.0	1.7	96.7	7.5	1,004.8
F60	11.7	55.4	31.8	20.6	2.1	1.8	116.7	9.1	1,049.3
IS23525	11.9	52.6	30.5	19.3	2.0	1.7	118.9	9.3	997.2
IS23536	13.7	51.7	32.2	17.2	2.0	1.8	136.7	10.7	1,025.0

FSY: fresh stalk yield; DSY: field dried stalk yield; JY: is a juice yield; CSY: conservative sugar yield; SY: sugar yield; SCJ: sugar concentration of juice; y: total soluble sugar; TEY: theoretical ethanol yield.

## Discussion

The agro-morphological diversity study of Chadian sweet sorghum showed significant variability in qualitative and quantitative characteristics. In fact, two main leaf midrib colors were observed; white and green which are also the main colors of this type of sorghum^28^. According to research by^28^, accessions with white midrib were not very juicy. However, our study showed that some white midrib accessions were very juicy. The sweet sorghum accessions from the Sudanese zone were identified to be from the *caudatum* and *bicolor* race, unlike the dry-season sorghum which are from the *durra* race^14^. According to^28^, sweet sorghum belongs to *bicolor*, *caudatum*, *durra* and hybrid *bicolor-guinea* race. Ritter et al.^33^ suggested that sweet sorghums are of polyphyletic origin, with relatives among *kafir*, *caudatum*, and other grain sorghum types.

Murray et al.^26^ identified three separate groups of sweet sorghum which often are classified together. He classified these major types as: syrup types; (historical and some modern) which were from the *caudatum* race, modern sugar and energy types; associated with the *kaffir/bicolor* races, and amber types; mainly *durra* and *bicolor* races.

Generally, the 0.5 t ha^−1^ average grain yield production in this study was quite low compared to the average 1.0 t ha^−1^ observed in Chadian grain sorghums or 0.87 t ha^−1^ for dry-season grain sorghum in the same region^14^. According to^7^, sweet sorghum is characterized by reduced grain yield as compared to grain sorghum. However, two of the accessions *Begon* (1.7 t ha^−1^) and *GWS lache* (1.5 t ha^−1^) showed higher than average grain yields and could be used to improve grain yield of sweet sorghum. Plant height, stem diameter, number of internodes, internode length and other morphological characteristics showed high variability in the accessions studied.

The brix from the current study differed significantly between accessions and it value ranged from 5.5 to 16.7%, with an average of 11.9%. Brix content was lower than that reported by^8,28^, who obtained brix value ranging from 8.9 to 21.8% and 11.8 to 22.5%, respectively. The "*Balnda*" and "*Chian Woua*" accessions had 16.7% brix, which was higher than what has been recorded in many sweet sorghum studies. According to^30^, optimal harvesting stage for sweet sorghum is when the juice contains 15.5–16.5% brix which is one of the most important characteristics necessary to obtain juice of high fermentable quality and thus maximize ethanol yield per hectare.

The moisture content of fresh stalk for the 105 landraces ranged from 33.7 to 74.4%, averaging 48.7%. The average value is lower than moisture contents obtained by^40^ (76.0%; using a single cultivar) and^10^ (81.0%; using three cultivars). But the value obtained on this current study were high than that obtained by^5^ (16.5%; using 73 sweet sorghum accessions). The higher end of the observed range of juice yield in this study were similar or higher than values obtained by^43^, but similar to values obtained by^41^ working with 31 sweet sorghum lines in Arizona.

Estimated sweet sorghum sugar yields in current study showed high level of diversity (*p* < *0.001*) amongst the 105 cultivars, the average value (2.2 Mg ha^−1^) being lower than estimated mean values (4.0 Mg ha^−1^ and 4.0 to10.7 Mg ha^−1^) reported by^38,41^; both evaluating 4–6 sweet sorghum lines at variable planting dates and locations respectively. However, 2.2 Mg ha^−1^ was higher than values reported by^34^ (1.8 Mg ha^−1^, 1 variety with variable NPK fertilization management),^44^ (1.8 Mg ha^−1^ in 2009, using 1 hybrid at variable N-fertilization rates) and^35^ (1.7 Mg ha^−1^ using 5 cultivars across 3 years). The average amount of theoretical ethanol yield (1,266 L ha^−1^) were lower than value (2,854 L ha^−1^) obtained by^41^, but higher than that obtained by^35^ (1,025 L ha^−1^) and^2^(1,000–1,149 L ha^−1^, using 12 cultivars). According to^19^, up to 13.2 Mg ha^−1^ of total sugars, equivalent to 7,682.0 L ha^−1^ of ethanol can be produced by sweet sorghum under favorable conditions.

The correlation matrix showed positive correlations of interest between Brix content with plant height, stem diameter, number of internodes and number of days to flowering. Brix is a measure of dissolved sugar to water mass ratio of a liquid; it was positively influenced by the maturity. According to^37^, all known sweet sorghums are tall, and prior research identified a positive correlation between height and sugar accumulation. The positive relation between brix and maturity suggest that early maturity may not be a desirable characteristics for sweet sorghum variety development since plants will need more days to accumulates more biomass and store energy in its stalk throughout the growing period^27,32,36^. Positive relations between plant height and days to flowering suggest that taller plants tended to flower later as observed by^8,42^. The study showed plant height, number of days to flowering and fresh stalk weight negatively affected the moisture content in fresh stalk. Similar results were obtained by^5^ working with sweet sorghum cultivars from the U.S. National Plant Germplasm System collection.

Yields of sugar and theoretical ethanol were significantly (*p* < *0.05*) and negatively influenced by potential grain yield and thousand grain weight. Indeed, sweet sorghum accessions are characterized by the accumulation of carbohydrates in their juicy stems^32^ to the detriment of the grains which remain rather poorly filled. Clerget et al.^4^ suggested a potential negative interaction between stem development and grain yield in sweet sorghum. This is contrary to grain sorghum accessions where accumulation of carbohydrates is done in favor of grains. Sweet sorghum landraces usually have small panicles and the stem sweetness is commonly attributed to low panicle strength^22^. However, the interactions and trade-offs between panicle size, grain filling and stem development are complex and can be complicated in photoperiod-sensitive sweet sorghums which tend to show great phenotypic plasticity. While competition for carbohydrates between grain filling and sugar storage in stems has been suggested by other studies^2,31^, the remobilization of stem reserves towards grain was frequently reported as being small^21^. Thus, varieties in current study which combined high stem reserves with comparatively good grain yield will be great candidates for dual-purpose (food-fuel) sweet sorghum breeding. These varieties are currently under consideration for a potential collaborative project between sorghum scientists in Chad and the United States. Such varieties were reported^25^ as being cultivated by farmers in semi-arid Mali.

## Conclusion

The current study showed that sweet sorghum accessions from Chad were from *caudatum* and *bicolor* race, and most of them had loose panicles with red seeds. Brix values ranged from 5.5 to 16.67% were found and greatly differed (*p* < *0.001*) among accessions. Two accessions "*Balnda*" and "*Chian Woua*" with a high brix value of (16.67%) were identified and could be used as source of sugar to improve grain sorghum in Chad. The yields of potential sugar and theoretical ethanol showed the values ranging from 0.45 to 5.3 Mg ha^−1^ and 279.5 to 3,101.2 L ha^−1^ respectively. The study showed high variability (*p* < *0.001*) for all assessed quantitative characters within sweet sorghum accessions from Chad, and identified four major groupings within accessions, each with multiple subgroupings except cluster 4 which had 2 accessions. This study provides valuable findings which could be used to improve sweet sorghum production in Chad for dual purpose use. Furthermore, the ten accessions with higher brix content than the five improved ICRISAT varieties could be used in biofuel (ethanol) breeding programs in similar geographical production regions. These accessions are currently been considered for improvement in a collaborative project with US based scientists.

## Supplementary information

Supplementary file 1.
