# Supplementary material for: Characterization of semi-arid Chadian sweet sorghum accessions as potential sources for sugar and ethanol production
Source: Sci Rep. 2020 Sep 11;10:14947. doi: 10.1038/s41598-020-71506-9 (PMC7486407; doi:10.1038/s41598-020-71506-9)
Supplement: Supplementary file 1 [file 41598_2020_71506_MOESM1_ESM.docx]

Supplementary Information

Manuscript Title: Characterization of semi-arid Chadian sweet sorghum accessions as potential sources for sugar and ethanol production.

Gapili Naoura^1^; Yves Emendack^2^*; Nébié Baloua^3^; Kirsten vom Brocke^4,7^; Mahamat Alhabib Hassan^1^; Nerbewende Sawadogo^5^; Amos Nodjasse Doyam^1^; Reoungal Djinodji ^1^; Giles Trouche^4,7^; Haydee E. Laza^6^.

^1^Institut Tchadien de Recherche Agronomique pour le Développement (ITRAD), B.P. 5400, N’Djaména, Chad.

^2^USDA-ARS, Cropping Systems Research Laboratory, Lubbock, TX.79424, USA.

^3^International Crops Research Institute for the Semi-Arid Tropics (ICRISAT), Mali.

^4^CIRAD, UMR AGAP, University of Montpellier, 34090, France.

^5^Université Joseph KI-ZERBO, Laboratoire Biosciences, Équipe Génétique et Amélioration des plantes, 03 BP 7021Ouagadougou 03, Burkina Faso.

^6^Texas Tech University, Department of Plant and Soil sciences, Lubbock, TX.79409, USA.

^7^CIRAD, INRA, UMR AGAPF-34398 Montpellier, France.

*Corresponding author, E-mail: [Yves.Emendack@ars.usda.gov](mailto:Yves.Emendack@ars.usda.gov); 3810 4^th^ Street Lubbock, Texas, United States. Telephone: +1 (806) 723-5224.

Supplementary Table 1: Quantitative traits accessed for semi-arid Chadian sweet sorghum.

| Acc. # | PHT | NDH | NDF | NIN | INL | PLL | PLW | PAL | PAW | FSW | DSW | SDI | PYI |
| --- | --- | --- | --- | --- | --- | --- | --- | --- | --- | --- | --- | --- | --- |
| 3 | 192.8 | 75.7 | 81.3 | 6.8 | 20.9 | 66.2 | 8.1 | 24.2 | 6.4 | 152.8 | 69.4 | 1.7 | 0.7 |
| 4 | 235.8 | 106 | 93.3 | 11.4 | 22.8 | 70.6 | 7.4 | 23.9 | 7.9 | 125.0 | 72.2 | 1.9 | 0.4 |
| 5 | 260.6 | 116.3 | 81.3 | 14.6 | 26.4 | 64.8 | 7.6 | 30.2 | 6.6 | 216.7 | 130.6 | 2.1 | 0.3 |
| 9 | 250.3 | 90.3 | 98.3 | 10.6 | 25.9 | 64.3 | 7.7 | 29.4 | 7.3 | 150.0 | 80.6 | 1.7 | 0.5 |
| 10 | 252.5 | 106.7 | 74.0 | 12.6 | 34.1 | 56.6 | 6.2 | 25.2 | 5.4 | 169.4 | 97.2 | 2.1 | 0.2 |
| 11 | 239.7 | 97.7 | 76.0 | 9.8 | 32.8 | 52.8 | 5.4 | 25.5 | 5.2 | 130.6 | 72.2 | 1.5 | 0.2 |
| 12 | 252.5 | 85.7 | 72.7 | 6.2 | 27.3 | 65.1 | 7.2 | 26.7 | 6.3 | 94.4 | 40.0 | 1.7 | 0.2 |
| 13 | 204.4 | 90.3 | 82.0 | 10.6 | 24.9 | 63.7 | 6.8 | 23.5 | 6.7 | 138.9 | 73.9 | 2.0 | 0.4 |
| 14 | 195.0 | 118.3 | 82.0 | 15.6 | 23.6 | 72.5 | 8.1 | 24.6 | 6.2 | 238.9 | 158.3 | 2.2 | 0.3 |
| 15 | 265.0 | 86.0 | 98.0 | 6.0 | 25.6 | 74.4 | 7.7 | 27.6 | 8.5 | 55.6 | 19.4 | 1.5 | 0.9 |
| 16 | 276.4 | 115 | 117.7 | 15.1 | 19.8 | 68.3 | 9.6 | 23.8 | 9.8 | 203.3 | 129.2 | 1.8 | 0.3 |
| 17 | 280.8 | 96.7 | 126.3 | 8.7 | 21.0 | 67.8 | 8.7 | 25.6 | 9.5 | 136.1 | 77.8 | 1.7 | 0.3 |
| 19 | 251.7 | 81.3 | 99.0 | 9.1 | 26.2 | 71.9 | 8.3 | 28.8 | 8.1 | 208.3 | 110 | 1.5 | 0.1 |
| 21 | 289.2 | 75.7 | 111.0 | 6.6 | 21.6 | 76 | 8.9 | 35.3 | 7.7 | 111.1 | 42.2 | 1.25 | 0.2 |
| 23 | 224.4 | 77.0 | 77.0 | 6.8 | 31.3 | 52.6 | 6.2 | 22.5 | 6.3 | 150.0 | 58.3 | 1.5 | 0.2 |
| 24 | 250.6 | 111.0 | 103.3 | 12.3 | 20.3 | 71.4 | 9.8 | 27.8 | 8.8 | 222.2 | 123.9 | 2.2 | 0.5 |
| 25 | 287.2 | 79.7 | 105.0 | 7.7 | 22.5 | 74.6 | 9.1 | 32.1 | 7.0 | 105.6 | 52.8 | 1.8 | 0.3 |
| 26 | 250.0 | 95.3 | 77.5 | 9.1 | 34.9 | 63.8 | 6.2 | 29.7 | 5.3 | 135 | 58.3 | 1.75 | 0.3 |
| 27 | 275.0 | 85.7 | 97.0 | 6.1 | 19.0 | 46.0 | 8.0 | 19.0 | 13.0 | 225.0 | 86.1 | 1.35 | -- |
| 28 | 225.6 | 87.3 | 76.7 | 6.8 | 27.4 | 59.1 | 6.2 | 29.2 | 6.4 | 194.4 | 88.9 | 1.5 | 0.3 |
| 30 | 252.5 | 76.3 | 77.0 | 6.4 | 28.9 | 67.8 | 7.6 | 25.4 | 8.2 | 72.2 | 32.2 | 1.6 | 0.5 |
| 31 | 231.1 | 78.0 | 88.0 | 7.5 | 22.6 | 71.4 | 7.1 | 24.7 | 10.4 | 91.7 | 34.2 | 1.6 | 0.5 |
| 32 | 168.9 | 85.3 | 78.7 | 10.0 | 21.8 | 65.1 | 9.3 | 22.3 | 6.8 | 122.2 | 61.1 | 1.6 | 0.3 |
| 34 | 236.4 | 102.7 | 97.3 | 12.8 | 21.4 | 82.8 | 8.0 | 26.4 | 6.6 | 128.9 | 76.7 | 1.9 | 0.3 |
| 36 | 262.8 | 90.3 | 104.3 | 11.6 | 18.9 | 72 | 10.3 | 25.4 | 9.7 | 144.4 | 72.2 | 2.0 | 1.5 |
| 42 | 275.8 | 91.3 | 111.3 | 10.2 | 20.4 | 70.1 | 8.2 | 31.7 | 7.1 | 147.2 | 77.8 | 1.6 | 0.6 |
| 43 | 247.8 | 77.0 | 96.7 | 6.6 | 24.4 | 69.9 | 7.8 | 23.1 | 6.5 | 141.7 | 52.8 | 1.0 | 0.4 |
| 44 | 238.1 | 102.0 | 119.7 | 13.3 | 20.6 | 68.3 | 9.2 | 17.1 | 7.5 | 191.7 | 116.7 | 2.1 | 0.4 |
| 45 | 296.4 | 92.7 | 103.7 | 8.7 | 24.2 | 74.0 | 8.3 | 33.4 | 7.5 | 155.6 | 77.8 | 1.6 | 0.8 |
| 46 | 297.5 | 115.7 | 119.0 | 13.8 | 20.7 | 67.8 | 10.1 | 21.7 | 7.8 | 213.9 | 132.2 | 2.1 | 0.4 |
| 47 | 237.8 | 113.7 | 92.0 | 14.2 | 19.1 | 73.4 | 8.3 | 21.4 | 7.7 | 161.1 | 97.2 | 2.1 | 0.4 |
| 49 | 230.3 | 105.7 | 96.0 | 12.4 | 18.7 | 70.1 | 8.1 | 19.2 | 6.4 | 133.3 | 74.4 | 1.9 | 0.8 |
| 50 | 267.5 | 72.0 | 93.0 | 5.8 | 23.0 | 67.0 | 8.2 | 28.8 | 7.4 | 97.2 | 37.2 | 1.3 | 0.3 |
| 51 | 215.6 | 86.3 | 106.0 | 9.3 | 19.8 | 71.1 | 9.1 | 22.2 | 8.4 | 238.9 | 127.8 | 1.6 | 0.8 |
| 53 | 128.9 | 111.0 | 69.3 | 13.4 | 19.4 | 57.9 | 7.0 | 16.7 | 6.6 | 186.1 | 108.3 | 2.2 | 0.7 |
| 54 | 264.4 | 98.0 | 99.0 | 12.9 | 22.1 | 76.1 | 8.6 | 26.1 | 8.2 | 143.3 | 88.9 | 2.0 | 0.9 |
| 55 | 249.2 | 95.0 | 95.7 | 9.4 | 21.5 | 75.1 | 7.3 | 25.7 | 7.3 | 130.6 | 65.6 | 1.5 | 0.5 |
| 56 | 192.2 | 82.0 | 90.0 | 6.3 | 18.8 | 71.1 | 9.1 | 23.6 | 6.6 | 308.3 | 133.3 | 1.5 | 0.8 |
| 58 | 256.9 | 103.0 | 117.0 | 12.5 | 16.6 | 66.1 | 8.6 | 22.8 | 8.4 | 181.7 | 104.4 | 2.2 | 0.5 |
| 60 | 217.2 | 115.0 | 107.0 | 14.7 | 14.4 | 64.9 | 9.1 | 21.3 | 7.3 | 180.6 | 73.9 | 1.9 | 0.6 |
| 61 | 261.4 | 111.3 | 91.3 | 13.6 | 19.4 | 68.6 | 9.5 | 30.6 | 8.1 | 147.2 | 86.1 | 2.0 | 0.6 |
| 63 | 290.3 | 88.0 | 98.0 | 8.6 | 28.1 | 65.1 | 8.9 | 23.2 | 7.6 | 86.1 | 50 | 1.3 | 0.2 |
| 64 | 263.1 | 76.7 | 101.3 | 7.5 | 19.5 | 63.0 | 9.2 | 28.5 | 8.3 | 283.3 | 127.8 | 1.4 | 0.6 |
| 66 | 222.5 | 77.5 | 115.0 | 6.8 | 16.2 | 62.5 | 9.6 | 18.2 | 9.1 | 170.8 | 75.0 | 1.2 | 0.5 |
| 68 | 296.1 | 119.7 | 113.0 | 13.8 | 22.7 | 76.1 | 9.8 | 31.8 | 7.5 | 180.6 | 121.7 | 2.0 | 0.7 |
| 70 | 227.3 | 98.0 | 125.7 | 13.6 | 16.1 | 63.9 | 8.8 | 16.2 | 8.4 | 172.2 | 86.1 | 2.0 | 0.4 |
| 71 | 228.9 | 126.3 | 124.3 | 14.1 | 17.7 | 67.7 | 9.2 | 22.7 | 8.4 | 202.8 | 100.6 | 1.9 | 0.4 |
| 72 | 294.2 | 79.7 | 115 | 7.8 | 19.7 | 76.6 | 9.4 | 27.6 | 8.2 | 169.4 | 75.0 | 1.5 | 0.5 |
| 75 | 298.3 | 89.7 | 116.3 | 8.2 | 21.2 | 64.5 | 8.1 | 31.1 | 7.6 | 94.4 | 42.8 | 1.7 | 0.6 |
| 77 | 165.6 | 94.3 | 97.0 | 9.4 | 14.6 | 74.5 | 9.3 | 21.4 | 6.9 | 122.2 | 77.8 | 1.5 | 0.3 |
| 78 | 134.2 | 96.0 | 86.0 | 11.7 | 19.9 | 66.9 | 7.4 | 10.9 | 5.0 | 161.1 | 83.3 | 1.7 | 1.7 |
| 80 | 294.7 | 71.7 | 111.3 | 8.2 | 24.2 | 83.0 | 8.6 | 33.6 | 8.7 | 233.3 | 102.8 | 1.6 | 0.5 |
| 81 | 294.7 | 85.0 | 104.3 | 12 | 24.4 | 83.3 | 8.7 | 35.8 | 8.8 | 58.3 | 36.7 | 1.8 | 0.5 |
| 82 | 242.2 | 97.3 | 103.0 | 9.1 | 17.3 | 72.8 | 8.9 | 28.3 | 9.7 | 119.4 | 81.7 | 1.7 | 0.5 |
| 83 | 277.3 | 119.0 | 115.0 | 13.3 | 18.2 | 68.2 | 8.7 | 26.8 | 8.0 | 141.7 | 88.3 | 2.0 | 0.4 |
| 84 | 215.8 | 108.0 | 91.0 | 16.2 | 25.2 | 71.3 | 7.0 | 20.7 | 5.3 | 135 | 75.0 | 2.2 | 0.5 |
| 85 | 250.0 | 109.3 | 81.7 | 13.9 | 27.9 | 56.4 | 5.9 | 24.4 | 5.2 | 168.3 | 91.7 | 2.1 | 0.2 |
| 86 | 278.6 | 90.0 | 110.3 | 7.3 | 20.6 | 72.4 | 7.9 | 29.7 | 6.6 | 369.4 | 144.4 | 1.8 | 0.3 |
| 87 | 284.6 | 73.3 | 95.7 | 4.9 | 28.3 | 75.1 | 7.0 | 26.0 | 7.6 | 161.1 | 61.1 | 1.3 | 0.5 |
| 88 | 163.9 | 110.0 | 88.3 | 14.4 | 18.8 | 68.6 | 8.2 | 22.2 | 6.8 | 155.6 | 77.8 | 1.9 | 0.5 |
| 89 | 173.9 | 73.3 | 77.0 | 5.8 | 20.9 | 67.1 | 7.7 | 18.1 | 7.0 | 113.9 | 37.2 | 1.4 | 0.9 |
| 91 | 219.2 | 92.0 | 86.0 | 8.7 | 21.8 | 66.1 | 7.2 | 25.7 | 7.1 | 141.7 | 82.2 | 1.8 | 0.3 |
| 92 | 129.3 | 119.7 | 80.0 | 14.7 | 17.5 | 54.2 | 7.3 | 21.6 | 6.6 | 208.3 | 122.2 | 2.1 | 0.7 |
| 94 | 208.5 | 68.3 | 91.0 | 5.3 | 23.0 | 70.2 | 7.9 | 25.4 | 7.0 | 150.0 | 40.6 | 1.3 | 0.2 |
| 95 | 195.1 | 78.3 | 88.3 | 7.8 | 23.7 | 62.2 | 9.0 | 18.9 | 5.9 | 213.9 | 122.2 | 1.5 | 0.5 |
| 96 | 141.9 | 82.0 | 73.0 | 9.1 | 19.7 | 56.4 | 7.4 | 21.3 | 6.1 | 208.3 | 110 | 1.5 | 0.5 |
| 97 | 163.5 | 125.7 | 79.7 | 12.45 | 21.1 | 68.9 | 7.0 | 20.0 | 7.0 | 138.9 | 85.6 | 1.8 | 0.3 |
| 98 | 217.2 | 81.0 | 119.0 | 7.1 | 14.8 | 70.1 | 10.2 | 19.1 | 8.4 | 133.3 | 75.0 | 1.3 | 0.9 |
| 99 | 269.4 | 90.3 | 116.3 | 7.8 | 16.2 | 71.3 | 8.9 | 22.1 | 8.1 | 123.3 | 57.2 | 1.6 | 0.5 |
| 100 | 226.7 | 104.3 | 94.3 | 14.6 | 20.3 | 71.6 | 7.6 | 24.7 | 8.1 | 220.6 | 122.2 | 2.3 | 0.5 |
| 101 | 156.4 | 103.0 | 75.3 | 14.9 | 20.0 | 73.6 | 7.8 | 23.2 | 5.3 | 166.7 | 97.2 | 2.0 | 0.8 |
| 102 | 163.6 | 88.3 | 78.7 | 8.8 | 21.2 | 62.8 | 7.9 | 21.3 | 6.6 | 161.1 | 91.1 | 1.8 | 0.7 |
| 103 | 167.2 | 123.7 | 77.0 | 15.2 | 21.9 | 60.4 | 7.3 | 22.2 | 4.7 | 131.7 | 77.8 | 1.8 | 1.1 |
| 104 | 175.7 | 96.7 | 86.3 | 12.6 | 21.8 | 64.2 | 7.8 | 22.9 | 4.9 | 180 | 102.8 | 2.0 | 1.0 |
| 106 | 145.8 | 94.3 | 72.0 | 10.3 | 18.5 | 56.6 | 7.8 | 22.8 | 6.3 | 144.4 | 84.4 | 1.6 | 0.5 |
| 107 | 135.6 | 110.3 | 68.3 | 12.2 | 19.7 | 55.9 | 6.9 | 14.8 | 4.9 | 136.1 | 77.8 | 1.9 | 0.8 |
| 108 | 201.9 | 111.7 | 95.0 | 16.2 | 19.7 | 74.7 | 8.3 | 26.3 | 8.2 | 225 | 126.7 | 2.3 | 0.6 |
| 110 | 230.0 | 73.0 | 81.7 | 6.8 | 22.6 | 62.9 | 8.2 | 24.4 | 7.4 | 322.2 | 116.7 | 1.3 | 0.4 |
| 112 | 291.9 | 97.3 | 112.3 | 9.6 | 18.7 | 71.2 | 8.7 | 32.8 | 10.5 | 136.1 | 71.1 | 1.8 | 0.6 |
| 113 | 271.1 | 77.0 | 121.7 | 6.1 | 18.7 | 75.4 | 9.3 | 24.8 | 8.7 | 119.4 | 50 | 1.5 | 0.8 |
| 114 | 291.1 | 97.0 | 113.7 | 10.2 | 20.1 | 66.4 | 9.2 | 26.7 | 9.3 | 127.8 | 63.9 | 2.2 | 1.2 |
| 115 | 232.5 | 104.3 | 82.0 | 11.6 | 23.3 | 69.4 | 8.3 | 27.0 | 8.2 | 216.7 | 119.4 | 2.1 | 0.3 |
| 116 | 298.3 | 102.3 | 108 | 13.5 | 23.3 | 73.1 | 9.5 | 31.2 | 8.2 | 197.2 | 101.1 | 2.0 | 1.0 |
| 117 | 283.1 | 110.3 | 107.3 | 14.8 | 21.9 | 72.3 | 8.6 | 27.9 | 5.7 | 175.0 | 103.3 | 2.2 | 0.5 |
| 118 | 210.7 | 115.0 | 77.3 | 15.2 | 19.5 | 58.8 | 7.9 | 24.2 | 7.6 | 238.9 | 140.0 | 2.1 | 0.2 |
| 119 | 218.1 | 81.7 | 91.0 | 8.4 | 18.8 | 82.9 | 9.6 | 22.7 | 7.3 | 366.7 | 169.4 | 1.5 | 0.7 |
| 120 | 185.8 | 100.3 | 81.0 | 13.8 | 22.6 | 68.0 | 8.7 | 20.8 | 8.9 | 119.4 | 61.1 | 2.1 | 0.9 |
| 121 | 148.3 | 78.7 | 74.0 | 6.9 | 22.1 | 60.1 | 6.6 | 23.8 | 4.9 | 105.6 | 41.7 | 1.4 | 0.8 |
| 123 | 166.4 | 91.7 | 76.3 | 10.8 | 19.4 | 64.3 | 8.5 | 22.6 | 7.2 | 127.8 | 78.3 | 1.7 | 0.7 |
| 124 | 180.3 | 105.3 | 82.7 | 12.2 | 20.4 | 60.1 | 7.0 | 20.2 | 5.7 | 138.9 | 76.7 | 1.8 | 0.8 |
| 125 | 170.3 | 80.0 | 78.0 | 5.7 | 20.6 | 66.4 | 7.7 | 22.1 | 6.7 | 38.9 | 15.6 | 1.3 | 0.88 |
| 126 | 242.2 | 93.3 | 92.7 | 7.8 | 22.1 | 70.1 | 7.3 | 23.6 | 5.7 | 69.4 | 38.9 | 1.2 | 0.3 |
| 127 | 155.8 | 76.3 | 77.7 | 8.2 | 18.2 | 60 | 7.4 | 20 | 5.1 | 308.3 | 138.9 | 1.6 | 0.8 |
| 129 | 291.7 | 117.7 | 96.7 | 14.5 | 25.3 | 74.3 | 9.0 | 30.6 | 6.9 | 122.2 | 78.3 | 2.1 | 0.64 |
| 130 | 266.7 | 124.3 | 114.3 | 15.1 | 17.7 | 64.7 | 8.9 | 21.2 | 7.6 | 147.2 | 83.9 | 1.9 | 0.24 |
| 131 | 290.0 | 101.7 | 105.3 | 13.3 | 21.9 | 75.3 | 8.3 | 33.4 | 7.4 | 173.3 | 94.4 | 2.0 | 0.5 |
| 132 | 265.8 | 76.7 | 103.0 | 8.8 | 18.2 | 72.1 | 9.7 | 25.3 | 8.8 | 222.2 | 105.6 | 1.5 | 0.7 |
| 133 | 149.4 | 80.3 | 78.7 | 7.1 | 18.5 | 64.7 | 8.8 | 19.4 | 5.8 | 200.0 | 81.7 | 1.1 | 0.5 |
| 135 | 277.8 | 69.3 | 123.7 | 4.9 | 21.2 | 65.3 | 8.3 | 22.8 | 8.3 | 122.2 | 36.1 | 1.5 | 0.28 |
| 136 | 270.3 | 76.0 | 111.7 | 7.3 | 18.2 | 71.1 | 8.7 | 31.2 | 9.8 | 87.5 | 45.8 | 1.2 | 0.6 |
| 137 | 296.9 | 101.3 | 105.0 | 13.9 | 22.6 | 71.2 | 9.8 | 30.2 | 10.3 | 175.0 | 100.0 | 2.2 | 0.36 |
| 139 | 247.8 | 95.0 | 94.3 | 10.2 | 20.7 | 69.9 | 7.1 | 23.6 | 8.4 | 150.0 | 77.8 | 1.7 | 0.48 |
| 140 | 260.6 | 94.3 | 97.7 | 11.0 | 23.6 | 82.8 | 7.4 | 24.3 | 7.1 | 119.4 | 61.1 | 1.6 | 0.6 |
| 141 | 245.8 | 78.7 | 94.3 | 5.8 | 23.9 | 78.6 | 8.3 | 23.4 | 8.1 | 152.8 | 63.9 | 1.4 | 0.64 |
| 142 | 295.6 | 114.3 | 101.3 | 14.3 | 24.7 | 75.6 | 8.3 | 35.9 | 9.8 | 194.4 | 125.0 | 2.2 | -- |
| F60 | 157.5 | 78.7 | 93.3 | 6.6 | 18.2 | 58.3 | 5.8 | 22.7 | 6.5 | 51.1 | 28.3 | 1.4 | -- |
| IS23525 | 186.9 | 76.3 | 87.3 | 5.8 | 17.8 | 69.1 | 11.4 | 21.1 | 6.9 | 177.8 | 58.3 | 1.2 | -- |
| IS23536 | 239.2 | 70.0 | 79.3 | 5.9 | 27.2 | 66.1 | 7.5 | 24.7 | 5.6 | 222.2 | 69.4 | 1.5 | -- |
| IS23541 | 213.3 | 95.0 | 88.3 | 10.9 | 19.2 | 82.7 | 10.7 | 24.3 | 7.6 | 133.3 | 73.3 | 1.7 | -- |
| IS23574 | 215.3 | 95.7 | 93.3 | 10.7 | 19.4 | 77.4 | 9.5 | 24.9 | 6.4 | 152.8 | 80.6 | 1.6 | -- |
| Average | 232.2 | 94.2 | 95.1 | 10.3 | 21.6 | 68.3 | 8.2 | 24.8 | 7.4 | 162.5 | 83.0 | 1.7 | 0.5 |

Acc.#; accession number, PHT; plant height, NDH; number of days to heading, NDF; number of days to flowering, NIN; number of internodes, INL; internode length, PLL; perultimate leaf length, PLW; perultimate leaf width, PAL; panicle length, PAW; panicle width, FSW; fresh stalk weight, DSW; dry stalk weight, SDI; stem diameter, PYI; potential yield.
